# Supplementary material for: Real-Life Study of the Benefit of Concomitant Radiotherapy with Cemiplimab in Advanced Cutaneous Squamous Cell Carcinoma (cSCC): A Retrospective Cohort Study
Source: Cancers (Basel). 2023 Jan 13;15(2):495. doi: 10.3390/cancers15020495 (PMC9856754; doi:10.3390/cancers15020495)
Supplement: Supplementary file 1 [file cancers-15-00495-s001.zip › cancers-2065307-supplementary.pdf]

cancers

MDPI

Table S1: Baseline characteristics per patient (part 1).

Patient	Group	Age (years)	Gender	ECOG Status	previous cScc (y/n)	Immuno depression (y/n)	Lymphopenia (y/n)	Staging	Site of metastasis	Site of primary tumour	size (mm)	previous lines of treatment	previous RT (Y/N)
1	C	74	M	2	yes	yes	yes	LacScc		face	20	no	no
2	C/RT	83	M	2	no	no	no	mcScc	LocoR	face	38	no	no
3	C/RT	88	M	1	yes	yes	no	mcScc	LocoR	cervical	30	no	no
4	C	81	F	2	no	no	yes	mcScc	LocoR	face		no	yes
5	C/RT	89	M	2	yes	no	no	mcScc	LocoR	cervical		no	no
6	C	86	M	1	yes	no	yes	mcScc	Distant	arm or leg	34	no	yes
7	C	75	M	1	yes	no	yes	mcScc	LocoR	scalp	20	no	yes
8	C	79	M	2	yes	no	no	mcScc	Distant	face	6	no	no
9	C	76	M	2	no	no	no	mcScc	LocoR	face	90	no	no
10	C/RT	89	F	2	no	no	yes	mcScc	LocoR	arm or leg	22	no	yes
11	C	80	M	2	yes	no	no	mcScc	Distant	face		no	yes
12	C	76	M	3	yes	no	no	mcScc	Distant	face	50	yes	yes
13	C/RT	87	M	1	yes	yes	no	mcScc	LocoR	face		no	no
14	C	69	M	1	no	yes	no	mcScc	LocoR	face	40	no	no
15	C/RT	78	M	1	yes	yes	yes	mcScc	LocoR	face	60	no	no

16	C	81	F	2	no	no	no	mcScc	LocoR	face		no	no
17	C	67	M	2	yes	yes	yes	mcScc	LocoR	face		no	yes
18	C	81	M	2	yes	no	no	mcScc	LocoR	face	40	no	yes
19	C	40	M	0	no	no	no	mcScc	LocoR	trunk		no	no
20	C	47	F	1	no	no	no	mcScc	Distant	face		yes	yes
21	C/RT	51	M	1	no	no	no	mcScc	Distant	arm or leg		yes	no
22	C	81	M	1	no	no	yes	mcScc	Distant	trunk		no	yes
23	C	80	M	1	no	no	no	mcScc	LocoR	face	40	no	no
24	C/RT	64	M	1	no	yes	yes	mcScc	Distant	face	30	no	no
25	C/RT	73	M	0	no	no	no	LacScc		face	20	no	no
26	C	90	M	2	yes	no	no	mcScc	LocoR	face	14	no	yes
27	C/RT	62	M	1	no	no	yes	mcScc	Distant	arm or leg	65	no	no
28	C	76	M	0	yes	no	no	mcScc	Distant	face	9	no	yes
29	C	84	F	1	yes	yes	yes	mcScc	LocoR	face		no	yes
30	C	82	M	1	yes	no	no	LacScc		face	27	no	yes
31	C/RT	76	M	2	yes	no	no	mcScc	Distant	trunk	120	no	no
32	C	73	M	2	yes	yes	no	LacScc		scalp	70	no	yes
33	C/RT	87	M	1	yes	no	yes	mcScc	LocoR	face		no	no

Baselines characteristics per patient (part 2)

Patient	degree of differentiation	PNI (y/n)	Bone erosion (y/n)	Invasion beyond subcutaneous fat (y/n)	Dose of cemiplimab	Intent of RT	Site of RT: primary tumour or metastasis	Site of RT	Dose/ fraction (Gy)	Nb of fraction	Prescribed dose (Gy)	BED (Gy)
1	well		no		3mg/kg/2w							
2	well		no		350 mg/3w	curative	metastasis	Cervical lymph nodes and parotid	3	23	69	89.7
3	moderate		yes		350 mg/3w	palliative	metastasis	Cervical lymph nodes	6	6	36	57.6
4	moderate	no	no	no	350 mg/3w							
5	poor		no		350 mg/3w	curative	metastasis	Parotid	6	2	12	19.2
6	well	no	yes	no	350 mg/3w							
7	moderate	yes	yes	yes	3mg/kg/2w							
8	well				350 mg/3w							
9					350 mg/3w							
10	poor		no	no	35 mg/3w	palliative	metastasis	Axillary node	6	6	36	57.6
11	well				350 mg/3w							
12			yes		350 mg/3w							
13	moderate				350 mg/3w	palliative	metastasis	Cervical lymph nodes	6	3	18	28.8
14	well		no	yes	both#							

15	well	no	no	no	350 mg/3 w	curative	metastasis $	1.Cervical lymph nodes	3	15	45	58.5
								2.Cervical lymph nodes	3	21	63	81.9
16	well	no	no	no	3mg/kg/2 w							
17	poor		yes		350 mg/3 w							
18	moderate	yes	no	yes	350 mg/3 w							
19	well		no	yes	3mg/kg/2 w							
20	well				350 mg/3 w							
21	well				350 mg/3 w	curative	Metastasis $	1.Inguinal nodes	2	35	70	84
								2.Subcutaneous nodule	6	7	42	67.2
22					350 mg/3 w							
23	well	no	no	no	350 mg/3 w							
24	poor	no	no	yes	350 mg/3 w	curative	Metastasis $	1.Cervical lymph nodes	2.4	29	69.6	86.3
								2. hepatic	2.4	24	57.6	71.4
25	well	no	no	yes	350 mg/3 w	curative	Primary tumour $	1.Primary tumour	1.8	35	63	74.3
								2.Primary tumour	2	35	70	84
26	moderate	yes	no	no	350 mg/3 w							
27	moderate	no	no	yes	350 mg/3 w	curative	Metastasis $	1.Inguinal nodes	6	2	12	19.2
								2.Sternum	3.7	8	29.6	40.55

28	moderate	no	no	no	350 mg/3 w							
29	poor		no	yes	Both#							
30	moderate	no	yes	yes	350 mg/3 w							
31	moderate	no	no	yes	350 mg/3 w	palliative	primary tumour	Primary tumour	6	2	12	19,2
32					3mg/kg/2 w							
33	well				350 mg/3 w	curative	metastasis	parotid	3	23	69	89,7

C: cemiplimab group; C/RT: cemiplimab/radiotherapy group; CSCC: cutaneous squamous cell carcinoma; LacScc: locally advanced cScc; mcScc: metastatic cScc; LocoR: locoregional; w: weeks; Gy : grey; BED: biologically effective dose. #: patients who received the two dosages; $ patients who received two radiotherapy sequences.

Table S2: Results per patient.

Patient	Group	BOR	DCR (y/n)	Time of follow-up (months)	Time of TTT (months)	Nb of cemiplimab cession (n=)	Time to response(months)	Event palliative care or death (y/n)	Positive clinical effect of RT(y/n)	Reason for discontinuation of cemiplimab	Disease status at cessation of cemiplimab	AE of cemiplimab (grade 0 to 5)	AE (type)	time to onset of AE (months)	AE (RT) (grade 0 to 5)
1	C	NE		4	4	8		yes		Palliative care decision or death	NE	0			
2	C/RT	CR	yes	17	17	25	3	no	yes	Loss of reimbursement for cemiplimab	CR	2	Rash maculo-papular	0,5	2
3	C/RT	SD	no	9	8	13		yes	yes	Palliative care decision or death	PD	3	fatigue	8	2

4	C	PR	yes	22	22	32	3	no		Loss of reimbursement for cemiplimab	PR	0	asymptomat ic gastritis		
5	C/R T	CR	yes	7	7	11	2	no	yes	Lost to follow-up	CR	1	colitis	3	0
6	C	PD		4	3	5		yes		Palliative care decision or death	PD	0			
7	C	CR	yes	32	32	51	17	no		Loss of reimbursement for cemiplimab	CR	3	pancreatitis	7	
8	C	NE		2	0	2		yes		Palliative care decision or death	NE	3	Chronic kidney disease	1	
9	C	PD		5	4	7		yes		Palliative care decision or death	PD	0			
10	C/R T	SD	no	7	6	9		yes	yes	Palliative care decision or death	PD	1	Chronic kidney disease	4	1
11	C	CR	yes	14	14	18	5	no		Loss of reimbursement for cemiplimab	CR	3	hepatic cytolysis	2	
12	C	NE		1	0	1		yes		Palliative care decision or death	NE	0			

13	C/RT	NE		1	1	1		yes	yes	Palliative care decision or death	NE	0			0
14	C	CR	yes	34	34	60	15	no		Loss of reimbursement for cemiplimab	CR	2	colitis	10	
15	C/RT	CR	yes	12	12	18	3	no	yes	Loss of reimbursement for cemiplimab	CR	0			1
16	C	NE		0	0	1		yes		Palliative care decision or death	NE	0			
17	C	PD		5	4	7		yes		Palliative care decision or death	PD	0			
18	C	CR	yes	7	7	11	3	no		Loss of reimbursement for cemiplimab	CR	2	hypothyroidism	4	
19	C	NE		3	3	4		yes		Palliative care decision or death	NE	3	fatigue	0	
20	C	PD		2	0	1		yes		Palliative care decision or death	PD	0			
21	C/RT	PD		9	3	4		yes	yes	Palliative care decision or death	PD	3	pancytopenia	3	2
22	C	NE		1	1	2		no		Lost to follow-up	NE	0			

23	C	CR	yes	20	20	29	3	no		Loss of reimbursement for cemiplimab	CR	2	asymptomatic lipase increase	8	
24	C/RT	PR	no	8	8	7	4	no	no	Loss of reimbursement for cemiplimab	PD	0			1
25	C/RT	CR	yes	10	10	15	3	no	yes	Loss of reimbursement for cemiplimab	CR	1	pruritus	7	1
26	C	PR	no	9	7	17	3	yes		Palliative care decision or death	PD	0			
27	C/RT	PD		10	6	5		yes	yes	Palliative care decision or death	PD	3	hyperglycemia	3	0
28	C	CR	yes	12	12	18	6	no		Loss of reimbursement for cemiplimab	CR	1	myalgia	12	
29	C	PR	yes	33	33	53	14	no		Loss of reimbursement for cemiplimab	PR	2	myalgia	17	
30	C	PR	yes	21	21	32	7	no		Loss of reimbursement for cemiplimab	PR	1	psoriasis	10	
31	C/RT	SD	no	7	4	5		yes	no	Palliative care decision or death	PD	0			0

32	C	SD	no	9	9	5	yes		Palliative care decision or death	PD	3	gamma-GT increased	1	
33	C/RT	SD	yes	7	7	11	no	yes	Loss of reimbursement for cemiplimab	SD	0			1

BOR: best overall response; CR = complete response; PR = partial response; SD = stable disease; PD = progressive disease; NE = non evaluated; DCR: disease control rate; C: cemiplimab group; C/RT: cemiplimab/radiotherapy group; TTT: treatment; NB: number; RT: radiotherapy; AE: adverse event (gradation from 0 to 5).
